# A Scalable and Accurate Targeted Gene Assembly Tool (SAT-Assembler) for Next-Generation Sequencing Data

**DOI:** 10.1371/journal.pcbi.1003737

**Published:** 2014-08-14

**Authors:** Yuan Zhang, Yanni Sun, James R. Cole

**Affiliations:** 1 Department of Computer Science and Engineering, Michigan State University, East Lansing, Michigan, United States of America; 2 Center for Microbial Ecology, Michigan State University, East Lansing, Michigan, United States of America; Heinrich Heine University, Germany

## Abstract

Gene assembly, which recovers gene segments from short reads, is an important step in functional analysis of next-generation sequencing data. Lacking quality reference genomes, *de novo* assembly is commonly used for RNA-Seq data of non-model organisms and metagenomic data. However, heterogeneous sequence coverage caused by heterogeneous expression or species abundance, similarity between isoforms or homologous genes, and large data size all pose challenges to *de novo* assembly. As a result, existing assembly tools tend to output fragmented contigs or chimeric contigs, or have high memory footprint. In this work, we introduce a targeted gene assembly program SAT-Assembler, which aims to recover gene families of particular interest to biologists. It addresses the above challenges by conducting family-specific homology search, homology-guided overlap graph construction, and careful graph traversal. It can be applied to both RNA-Seq and metagenomic data. Our experimental results on an *Arabidopsis* RNA-Seq data set and two metagenomic data sets show that SAT-Assembler has smaller memory usage, comparable or better gene coverage, and lower chimera rate for assembling a set of genes from one or multiple pathways compared with other assembly tools. Moreover, the family-specific design and rapid homology search allow SAT-Assembler to be naturally compatible with parallel computing platforms. The source code of SAT-Assembler is available at https://sourceforge.net/projects/sat-assembler/. The data sets and experimental settings can be found in supplementary material.

## Author Summary

Next-generation sequencing (NGS) provides an efficient and affordable way to sequence the genomes or transcriptomes of a large amount of organisms. With fast accumulation of the sequencing data from various NGS projects, the bottleneck is to efficiently mine useful knowledge from the data. As NGS platforms usually generate short and fragmented sequences (reads), one key step to annotate NGS data is to assemble short reads into longer contigs, which are then used to recover functional elements such as protein-coding genes. Short read assembly remains one of the most difficult computational problems in genomics. In particular, the performance of existing assembly tools is not satisfactory on complicated NGS data sets. They cannot reliably separate genes of high similarity, recover under-represented genes, and incur high computational time and memory usage. Hence, we propose a targeted gene assembly tool, SAT-Assembler, to assemble genes of interest directly from NGS data with low memory usage and high accuracy. Our experimental results on a transcriptomic data set and two microbial community data sets showed that SAT-Assembler used less memory and recovered more target genes with better accuracy than existing tools.

This is a *PLOS Computational Biology* Methods article.

## Introduction

Advances in next-generation sequencing (NGS) technologies enable sequencing of transcriptoms of a large number of non-model organisms (RNA-Seq) and species from various environmental samples (metagenomic data). Functional annotation is an important step in analyzing these NGS data sets. For RNA-Seq of non-model organisms and metagenomic data, which lack quality reference genomes, a commonly used functional annotation pipeline conducts *de novo* assembly first and applies functional annotation analysis to the assembled contigs. This pipeline has been widely adopted in functional analysis of RNA-Seq data [1]–[4] and gene-centric metagenomic analysis [5]–[9]. One of the most informative and practical steps in functional analysis is homology search, which assigns functions to contigs using significant matches against non-redundant protein sequence databases or secondary databases including protein families, domains and functional sites. The performance of downstream functional analysis largely depends on the quality of the *de novo* assembly, which is still a challenging problem for RNA-Seq and metagenomic data.

The problem of assembling NGS reads has received considerable effort [10], [11]. Different programs have been developed for transcriptome assembly [12]–[17] and metagenomic assembly [18]–[24]. *De novo* RNA-Seq assembly aims to identify transcribed genes, separate isoforms, and quantify gene expression while metagenomic assembly intends to recover individual genomes from environmental samples. Often there are specific sets of genes in pathways that are of special interest, for example carbon and nitrogen cycling pathways in the response of permafrost to global warming [25] in soil metagenomic data, while much of bulk *de novo* assemblies consist of less interesting "housekeeping genes" and genes with unknown function that give little insight to the specific question at hand. Thus, gene-centric analysis using homology search is a direct and effective strategy to study complicated transcriptomic and metagenomic data.

*De novo* gene assembly for RNA-Seq and metagenomic data share similar challenges in algorithm design. First, genes that have divergent expression levels in RNA-Seq data or highly different abundance in metagenomic data lead to a wide range of sequence coverage. Even worse, the sequence coverage along the same gene can be highly different due to bias in sequencing protocols. Using one set of assembly parameters for all genes or even for different regions of the same gene leads to unsatisfactory performance, resulting in either fragmented contigs or chimeric contigs. Although methods using multiple *k-mer* sizes have been developed to identify transcripts in a broader coverage range, the choice of *k-mer* size is still ad hoc. Second, expression of gene isoforms due to alternative splicing (AS) events and expression of genes with shared short identical sequence regions in RNA-Seq data as well as the high similarity of orthologous genes in metagenomic data grossly compound *de novo* assembly. Chimeric contigs tend to be produced between highly similar transcripts or gene homologs. Third, existing assembly programs suffer from either long computational time or high memory usage or both. New developments in sequencing technology such as today's Illumina HiSeq can produce up to 150 bp 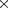 180 million reads of a total of 27 Gbp in a single run in single-end mode. Using all seven lanes can output up to 189 Gbp in one run and close to double that in paired-end mode. Two popular RNA-Seq assembly programs, Trinity and Oases, incur the longest running time and the maximum memory usage in comparison with other assembly tools [26], respectively. Using small *k-mer* size in Osases may require resources exceeding the memory space of a typical computing server on existing data [26]. Thus, scalable methods and tools are urgently needed to analyze new sequencing data.

To improve gene assembly in RNA-Seq and metagenomic data that lack quality reference genomes, we propose a different scheme, which conducts homology search first and then family-specific gene assembly. The proposed assembly method takes genes of interest as input, which can include specific sets of genes in pathways that are of special interest or well-characterized protein or domain families in existing databases. Note that although we conduct homology search for reads, assembly is still indispensable for functional analysis because the preferred products for a majority of users are relatively complete genes.

The main novelties of our method and how those are used to address the above challenges are summarized below. First, by conducting homology search on reads directly, all reads will be first classified into different gene families. *De novo* assembly will only be conducted for reads in each family. The input size for assembly is significantly decreased compared to bulk assembly. Second, the alignments output by homology search are used to guide family-specific gene assembly. We propose a novel overlap graph construction algorithm that can achieve maximum connectivity and accuracy for genes of both low and high sequence coverage. Third, we use a modified depth-first-search (DFS) that carefully incorporates multiple information including paired-end reads, transitive edges, and coverage information to guide graph traversal. The novel graph construction algorithm together with the carefully designed traversal algorithm can maximumly avoid generation of chimeric contigs.

### Related work

As gene families of interest are used as input, our algorithm employs homology search against gene families in assembly graph construction. Using genomes or proteome of related species to boost and optimize genome assembly has been proposed or implemented in a group of assembly programs [23], [24], [27]–[35]. The contigs belonging to a single gene or a block of genome in the related species are ordered, oriented, and assembled. Most of these programs are designed to improve genome assembly. A few of these comparative or gene-boosted assembly programs are specifically designed for RNA-Seq or metagenomic assembly. For example, Surget-Groba et al. [30] carefully considered the highly heterogeneous sequence coverage of transcripts and employed multi-k and proteome of a related species to optimize transcriptome assembly. Dutilh et al. [29] used one closely related reference genome to increase assembly performance of microbial genomes in metagenomic data. Ye's group used homologous genes to stitch gene fragments for gene assembly in metagenomic data [24].

Our work is different from existing comparative assembly approaches in the following aspects. First, our tool does not require any related species as input. Most of the existing comparative approaches are limited by the availability of closely related reference genomes. Low similarity between the to-be-assembled genes or genomes and the related genes or genomes can lead to wrongly assembled contigs. Our tool uses well-characterized genes of particular interest or ubiquitously represented sequence families such as those from family databases of proteins, domains, or functional sites as input to guide assembly. Second, as we use sequence families rather than a single sequence as reference, profile-based alignment methods rather than pairwise sequence alignment or exact sequence mapping are applied to conduct homology search. Profile-based methods tend to be more sensitive for remote homology search. Third, to our best knowledge, SAT-Assembler is the first tool that uses consistency between sequence overlap and alignment overlap for edge creation in an overlap graph.

Another type of RNA-Seq assembly tool, Cufflinks, assembles gene isoforms due to alternative transcription and splicing and improves transcriptome-based genome annotation [17]. Cufflinks and SAT-Assembler require different types of input and are targeted at different applications. Their major differences are summarized below. First, Cufflinks needs the reference genome as input while SAT-Assembler uses sequence families as input. The input families do not need to contain any genomic sequence or protein products from the reference genome. Instead, they may contain a large number of gene or protein sequences from other species with variable evolutionary distances. For example, the average sequence identity of protein (domain) families in Pfam ranges from 20% to over 90%. The conservation and evolutionary changes of the member sequences are summarized into a profile HMM, which can share high or low similarity with the genes in the reference genome. Second, Cufflinks and SAT-Assembler have different applications. Cufflinks is used to annotate transcripts and gene isoforms for species with known reference genomes. SAT-Assembler is applied to RNA-Seq data of non-model organisms or metagenomic data, which do not have reference genomes. Third, although both tools conduct sequence alignment between reads and the reference genomes or sequence families in the first step, the alignment algorithms are highly different. The read alignment in Cufflinks relies on read mapping tools such as TopHat [36] and Bowtie [37], which allow only minor changes caused by, for example, sequencing errors. On the other hand, the profile HMM-based alignment in SAT-Assembler can handle a large number of evolutionary changes including substitutions, insertions, and deletions.

## Methods

### Overview of SAT-assembler

**Figure 1:**
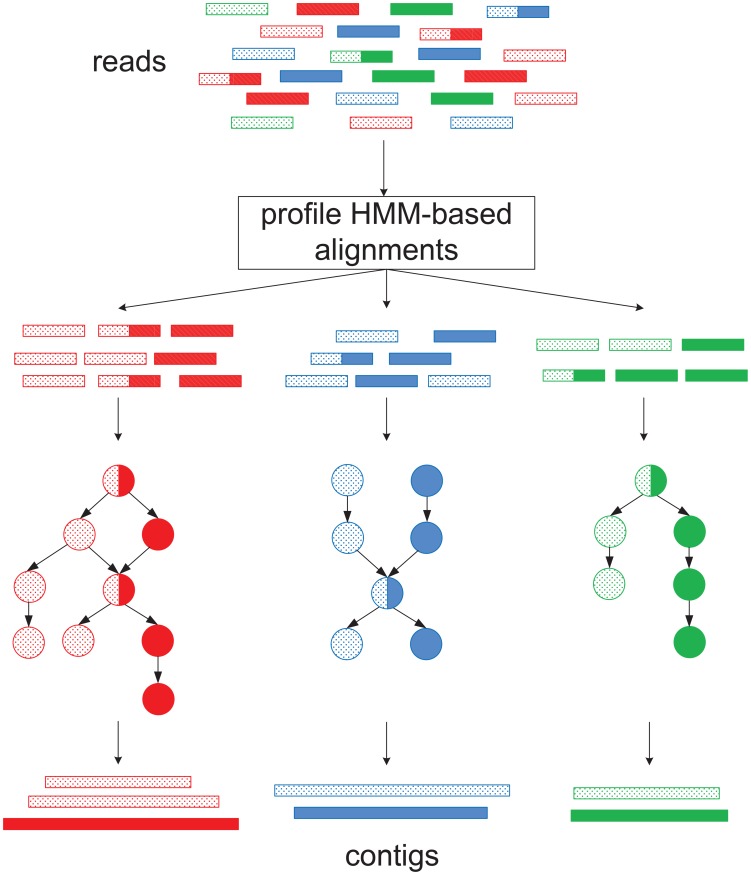
The pipeline of SAT-Assembler. Reads of the same color belong to the same gene family. Reads from different genes of the same family are distinguished using different patterns. Reads shared by multiple genes from the same family have multiple patterns.

Our tool can be divided into two main stages. First, we align reads against profile hidden Markov models (HMMs), which effectively represent the underlying gene families. This stage classifies the whole input data set into subsets of reads sequenced from different gene families. Second, SAT-Assembler constructs a family-specific overlap graph and assembles reads from the same family into contigs using a graph traversal algorithm. The graph construction is supervised by the alignment information from the first stage and aims to obtain maximum connectivity between reads while avoiding false connections. In particular, it can accurately capture small overlaps between reads from lowly sequenced regions and improves the assembly of lowly transcribed or encoded genes. The graph traversal is guided by multiple types of information to avoid generation of chimeric contigs. Finally, paired-end reads are used to scaffold contigs from the same genes into *super contigs*, which are sets of contigs that are from the same scaffolds. Fig. 1 is a schematic representation of the pipeline of SAT-Assembler.

### Profile HMM-based homology search

Our method conducts homology search on reads first. Depending on the algorithms and the target databases, homology search methods can be divided into two types. The first type compares the sequences against protein sequence databases using pairwise alignment tools such as BLAST [38]. The second type uses profile-based homology search to classify queries into characterized protein domain or family databases such as Pfam [39], [40], TIGRFAM [41], FIGfams [42], InterProScan [43], etc.

Applying profile-based homology search to NGS reads has several advantages. First, the number of gene families is significantly smaller than the number of sequences, rendering a much smaller search space. For example, there are only about 13,000 manually curated protein families in Pfam. Together these cover nearly 80% of the UniProt Knowledgebase and the coverage is increasing every year as enough information becomes available to form new families [40]. As the profile-based homology search tool HMMER is as fast as BLAST [44], using profile-based search provides a shorter search time. Second, previous work [45] has shown that using family information can improve the sensitivity of remote protein homology search. For the transcriptomes of non-model organism and metagenomic data, sensitive remote homology search is especially important for identifying possible new homologs. Third, although short reads can pose challenges to both types of homology search [46], [47], empirical studies on thousands of families [47] showed that the performance of profile-based homology search improved quickly with increasing read size. For a read length of 85 bases, the sensitivity is close to 1.0 for moderately and highly conserved domains. Thus, for read lengths produced by modern NGS technologies, profile-based homology search methods are capable of classifying many reads into their native families with high specificity.

SAT-Assembler aligns query reads against input families using HMMER with the default E-value threshold 10. Reads that generate HMMER hits are classified into the corresponding family and fed into the next stage. In most cases, a read can only be classified into a single family. However, because some input families share similarities, some reads may be classified into multiple input families. In practice, we only classify a read into at most three families with the three smallest E-values.

### Alignment-informed graph construction

The first stage not only classifies query reads into their native families but provides important alignment information for *de novo* assembly. The alignment positions are used to guide overlap graph construction. A standard overlap graph is defined as 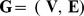, where each non-duplicate read is a node and an overlap larger than a given cutoff is indicated by a directed edge. Our overlap graph is different from a standard overlap graph [48]–[50] in the edge creation criteria and graph construction procedure.

**Figure 2:**
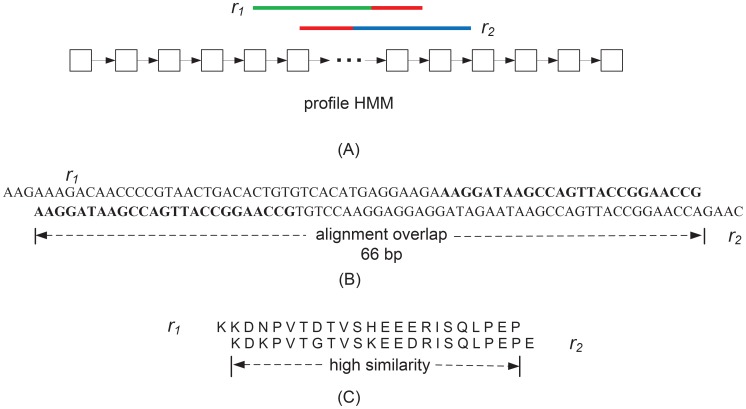
Alignment overlap and sequence overlap. (A) Two reads 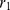 and 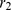 sequenced from different genes of the same family are aligned to the profile HMM of the family. Their sequence overlap is indicated in red. (B) Read 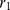 and read 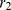 have an alignment overlap of 66 and a sequence overlap of 25 (in bold). (C) Sequenced from homologous genes, 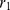 and 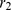 show higher similarity in their translated peptide sequences than in their DNA sequences.

In a standard overlap graph, edge creation only depends on the sequence overlap, which is not ideal for genes of heterogeneous sequence coverage. We add edges by considering the relationship between two types of overlaps: alignment overlap and sequence overlap. As HMMER outputs alignments represented by amino acids, all overlaps are converted into the unit of bp for consistency. For simplicity of explanation, a read 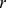 corresponds to vertex 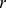 in G. For two reads 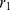 and 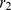, an edge is created from the corresponding node 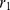 to node 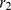 if the following criteria are satisfied: i) the alignment position of 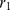 is smaller than 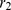; ii) the alignments of 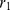 and 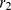 overlap by at least 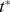, a user-defined threshold; iii) the sequence overlap of the two reads is consistent with the overlap in their alignment positions. Suppose 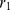 aligns to the model between 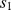 and 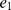, and 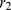 aligns to the model between 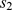 and 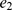, where 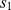 and 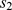 are alignment starting positions in the model and 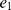 and 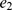 are alignment ending positions in the model. The alignment overlap is the number of bases converted from the number of amino acids in the overlapping region between 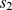 and 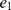. For example, the overlapping region between 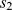 and 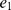 in Fig. 2.(C) contains 22 amino acids, which are converted into 66 bases of alignment overlap. Criterion 3 is the key observation to connect reads that are sequenced from the same gene rather than from orthologous or paralogous genes because the latter can have very different sequence and alignment overlaps. An example is given in Fig. 2, in which read 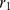 and read 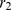 are from two homologous genes. Their sequence overlap and alignment overlap are 25 and 66 respectively. Other assembly tools such as Trinity will create an edge between 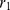 and 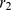 when the *k-mer* size is 25. However, because their alignment overlap and sequence overlap are inconsistent, SAT-Assembler does not connect them, avoiding a wrong connection between reads from homologous genes.

The consistency-based edge creation also allows us to improve connectivity in regions with low sequence coverage. For relatively small overlaps, we still allow an edge if the alignment overlap and sequence overlap are similar to each other. The intuition is that the chance that reads with random overlaps can be aligned to the same model with similar alignment overlap is small.

To quantify the consistency between the two types of overlaps, we introduce the *relative overlap difference* defined by 
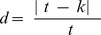
, where 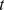 is alignment overlap and 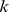 is sequence overlap. Criterion 3 is satisfied only when 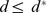, where 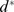 is a predefined cutoff with a default value of 0.15. We examined relative overlap difference in both real RNA-Seq and metagenomic data sets. For reads sequenced from the same gene and from different genes, the average relative overlap differences are 0.072 and 0.89 respectively. To avoid small random overlaps, we use 20 as the default value for 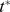, the alignment overlap threshold.

Our overlap graph construction is different from standard overlap graph construction in that it does not need all-against-all sequence comparison. We first sort the reads by their alignment positions in a non-decreasing order. We only check the sequence overlap between two reads if their alignment overlap passes 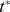. Therefore, the alignment information increases the efficiency of graph construction (running time analysis can be found in the section of Running Time Analysis). To incorporate substitution sequencing errors introduced by some NGS platforms, we allow a certain number 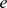 of mismatches in the sequence overlap. That is, the overlap between two hits 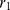 and 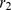 is the longest suffix of 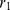 that has a Hamming distance 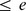 to a prefix of 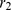. In our current implementation, 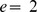. The parameter 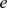 can be adjusted to fit the error rate of the input data.

### Pruning and optimization of overlap graphs

Transitive edges correspond to edges whose two end nodes are connected by an alternative path (usually with higher coverage). They add unnecessary edges without contributing to the connectivity of the graph and are removed before *de novo* assembly. Before removing them, SAT-Assembler keeps a record of all the pairs of nodes connected by transitive edges because a transitive edge indicates that a pair of nodes are from the same gene region. This information will be used to guide the graph traversal.

If a node has only one outgoing edge that points to another node that has only one incoming edge these two nodes can be merged as one node. Tips are identified and removed using the topology-based pruning methods as in Velvet [11].

Although our edge creation method excludes most random sequence overlaps, some erroneous edges still exist. An edge is highly likely to be erroneous if it is inferior to another edge that shares a head node or tail node with it. An edge 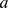 is inferior to another edge 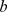 if the following two criteria are met: i) the sequence overlap of 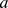 is smaller than half of that of 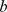; ii) the Hamming distance of sequence overlap of 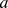 is larger than that of 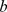. A random overlap is more likely to be much smaller and have more mismatches than a true overlap. Therefore, these two criteria will help us remove most erroneous edges.

### Guided graph traversal using multiple types of information

**Figure 3:**
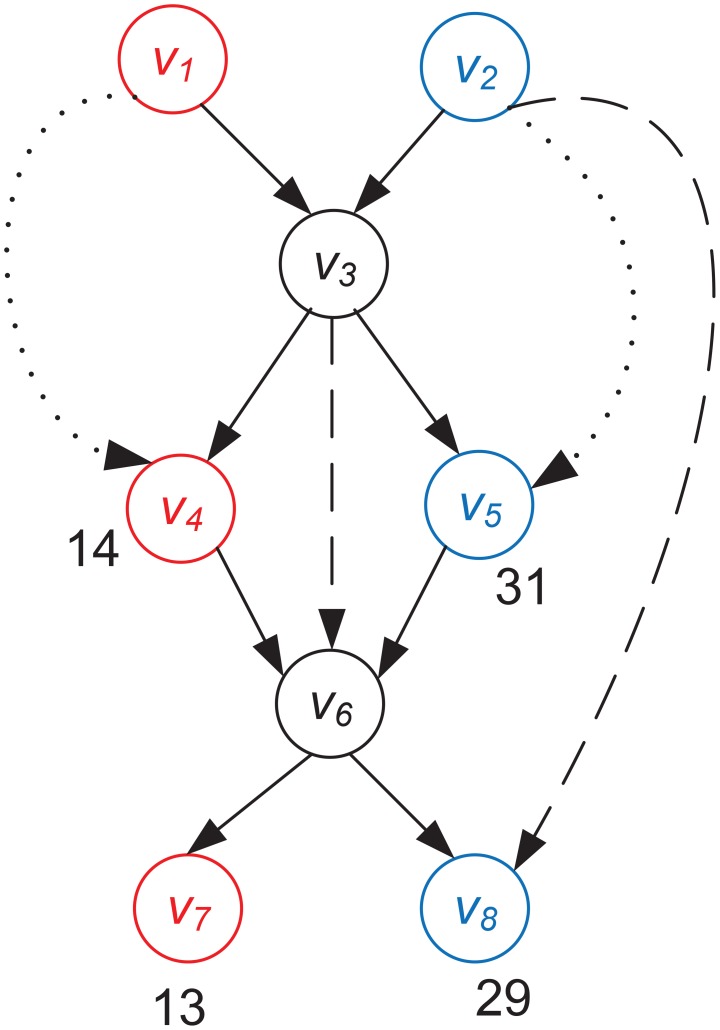
Graph traversal using multiple types of information. A graph containing reads from two different genes 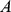 and 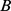. Nodes in red (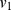, 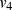, and 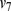) and in blue (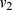, 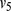, and 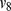) are from gene 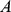 and gene 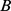 respectively. Nodes in black (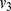 and 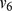) are chimeric nodes because they are shared by the two genes. Arrows with solid lines are real edges. Arrows with dotted lines and dashed lines indicate transitive edges and paired-end reads between two nodes respectively.

Once a family-specific graph is constructed and optimized, the goal is to conduct a graph traversal to output paths corresponding to genes. The traversal starts with nodes without incoming edges. The challenge arises when two or more genes contain a common or similar subsequence, leading to *chimeric nodes* such as 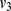 and 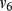 in Fig. 3.

Chimeric nodes add to the complexity of the graph traversal by leading to chimeric contigs. For example, the path 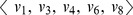 contains nodes exclusively from both genes and thus is a chimeric path. SAT-Assembler employs three types of information to guide the graph traversal to recover correct gene paths: transitive edges, paired-end reads, and coverage. We describe the key steps of our graph traversal algorithm using Fig. 3. The goal is to output two correct paths corresponding to the two genes.

A paired-end read represents two reads appearing in the same genome with known order (by our homology search) and distance range (insert size). Although transitive edges are removed at the stage of graph pruning they can act as a paired-end read with a small insert size. Therefore, both transitive edges and paired-end reads can be used to examine whether two nodes are from the same gene. Two nodes that are not connected by an edge are said to have *supports* or be *supported* if there are transitive edges or paired-end reads between them. For paired-end read supports, we further require that their distance in the path be consistent with the insert size.

Different from previous traversal algorithms, we divide supports into two types, *critical supports* and *non-critical supports*. Critical supports can be used to resolve branching in graph traversal while non-critical supports are not able to distinguish different gene paths. For example, a graph traversal generates a path 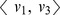. The node 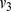 has two outgoing edges 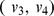 and 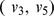. If there is a support between 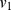 and 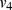, such as the transitive edge in Fig. 3, the traversal will be guided to visit 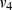 in next step. This transitive edge provides a critical support for correct traversal. However, the support between 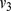 and 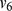 is not "critical" for guiding the graph traversal because any path that has visited 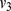 needs to visit 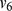. In Fig. 3, the support between 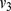 and 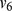 is a non-critical support while all the other supports are critical supports.

When there is no support between two non-chimeric nodes, *node coverage* will be used to resolve the branches. The coverage of a node is the total size of reads normalized by the length of the assembled sequence of the node. For protein-coding genes, although the sequence coverage is usually not uniform along the genes its change is gradual rather than sharp. Thus, the coverage of two consecutive non-chimeric nodes in the same path should reflect this observation. Any sharp change indicates a wrong path. For example, in Fig. 3, 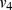 and 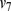 have similar coverage, as do 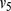 and 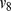. 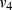 and 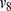, however, have significantly different coverage. Therefore, a path that has visited 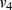 and 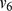 should next visit 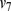 instead of 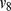.

We use a bounded depth-first search (DFS) algorithm to generate correct paths. While a typical DFS takes exponential time to generate all simple paths between two nodes, our graph traversal method makes use of critical supports to bound the search and only visits the correct paths, effectively reducing the time complexity of path generation. During search, we will proceed to those successors of the current node that provide critical supports. If none of the successive non-chimeric nodes has supports with any of the previously visited non-chimeric nodes, we will proceed to the one that has a similar coverage to the recently visited non-chimeric node given that their coverage is similar enough. Otherwise, it is highly likely that the current node is not from the same gene as any of its successors. Therefore, we will output the current path and start a new path from its successors. The traversal stops when there is no appropriate successive node available. All paths with critical supports above a given threshold will be output.

### Contig scaffolding

Assembly tools may output multiple contigs from the same gene. There are two main reasons for the fragmentation: i) some regions between contigs are not sequenced due to sequencing bias, PCR bias, low transcription level or abundance; ii) reads from lowly conserved regions of the gene may not pass the homology search and thus are not used to construct the graph. The contigs are oriented and connected using their alignment positions against the underlying profile HMM and paired-end reads. The scaffolding results in super contigs.

### Distinguish gene isoforms due to alternative splicing

**Figure 4:**
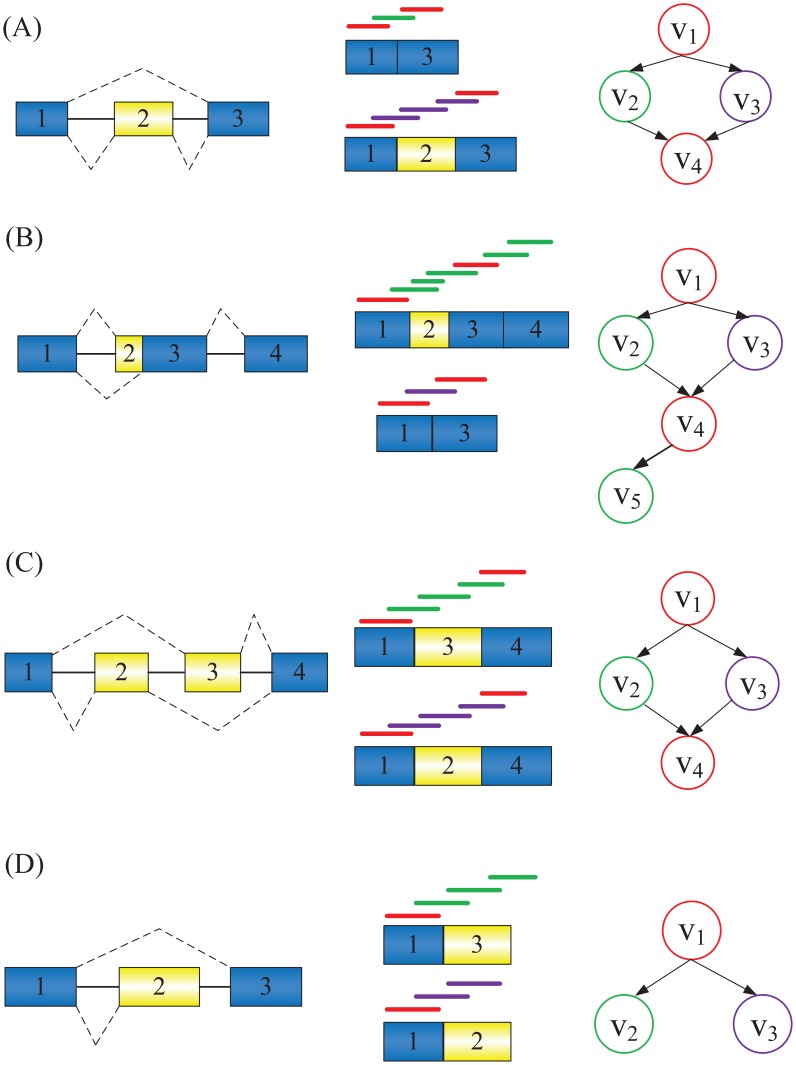
Distinguish gene isoforms due to alternative splicing. Overlap graphs constructed from different groups of AS events. Each case consists of the isoforms due to AS events (left), the positions of reads from the isoforms (middle), and the overlap graphs constructed from these isoforms (right). Constitutive exons are shown in blue and alternatively spliced regions in yellow. Introns are represented by solid lines, and dashed lines indicate splicing options. In the middle panel of each case, the red lines represent reads sequenced from exons shared by both isoforms. The green and purple lines represent reads that are exclusively sequenced from each isoform. In the right panel of each case, the red nodes represent reads sequenced from exons shared by both isoforms. The green and purple nodes represent reads that are exclusively sequenced from each isoform. Cases (A), (B), (C), and (D) represent four different groups of AS events.

SAT-Assembler can distinguish not only different target genes but also gene isoforms caused by AS events. Here we classify the seven different types of alternative splicing events [51] into four different groups. Fig. 4 shows how overlap graphs are constructed for these four groups of AS events. Each of the group is represented by one AS event in [51]. All the other types fall into one of these groups.

In Cases (A), (C), and (D) of Fig. 4, different isoforms correspond to different paths of the overlap graphs. Therefore, isoforms generated by AS events from these groups can be distinguished by generating contigs from these paths. In Case (B), there are two paths that begin with a root node and end with a sink node: 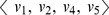 and 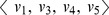. The first path recovers the first isoform and the second path corresponds to a chimeric contig. However, reads in 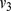 and 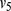 are not from the same isoform. Therefore, there will be no paired end support between them. Our graph traversal algorithm will stop in node 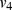 without proceeding to node 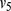. Therefore, SAT-Assembler can still correctly recover both isoforms in this case. In practice, different types of alternative splicing events can occur together, further compounding the assembly. In these cases, SAT-Assembler relies on multiple types of information such as paired end reads, transitive edges, and coverage to distinguish different isoforms, as described in the section of Guided Graph Traversal Using Multiple Types of Information. The performance of assembly tools on distinguishing gene isoforms can be found in the section of Performance of Recovering Gene Isoforms.

### Running time analysis

Let the number of input reads be 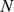 and the average read length be 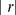. The time complexity of the homology search stage is 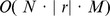 for one input family, where 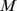 is length of the profile HMM and 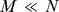. Suppose 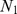 reads have passed the homology search stage. Usually, 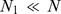. The time complexity of graph construction is 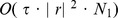, where 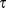 is the average number of overlapping alignments longer than a given cutoff. During graph construction, we use alignment positions to guide the overlap computation, avoiding the all-against-all comparison needed in a standard overlap graph construction. The time complexity of graph traveral is 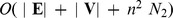, where 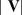 is the number of nodes, 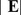 is the number of edges, 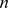 is the number of read pairs that have critical supports, and 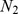 is the number of correct paths in the graph. The time complexity of the scaffoding stage is 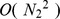. Because of various optimization techniques and heuristics, the latest version of HMMER is as fast as BLAST [44]. Considering 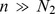, the time complexity of scaffolding is much smaller than graph traversal. Therefore, the overall running time is determined by the graph traversal stage.

## Results

To show the utility of SAT-Assembler, we applied it to an *Arabidopsis* RNA-Seq data set and two metagenomic data sets. The first metagenomic data set was sequenced from highly diverse *bacterial* and *archaeal* synthetic communities and the second data set was sequenced from human gut microbial communities. We compared the completeness and correctness of assembly, length of contigs, memory usage, and running time of different *de novo* assembly tools, including SAT-Assembler, Velvet, Oases, Trinity, IDBA-Tran, Trans-ABySS, IDBA-UD, and MetaVelvet.

**Figure 5:**
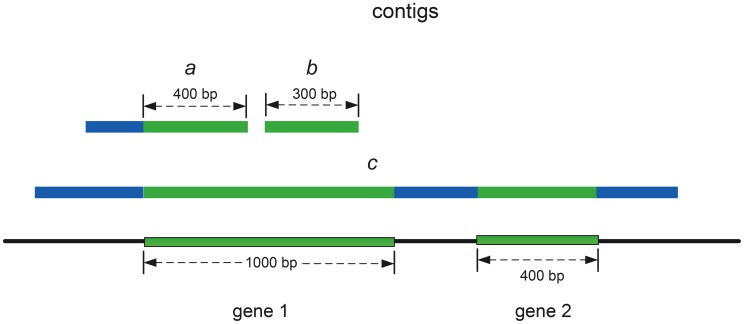
A schematic representation of gene segments. Three contigs and their gene segments. Gene 1 and gene 2 are target genes. Contigs 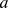 and 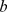 each contain one gene segment (green parts). Contig 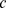 contains two gene segments (green parts). The blue parts of the contigs are not gene segments.

For targeted gene assembly, we are interested in evaluating the performance of each tool in recovering the target genes. Thus, for bulk assembly tools which output contigs containing multiple genes and intergenic regions, we first extract *gene segments* from the contigs. Fig. 5 shows examples of gene segments inside contigs. In each contig, only segments overlapping target genes are used for performance evaluation. For all experiments, by mapping reads to an annotated reference genome or characterized genes from existing databases, we constructed a set of reference/target genes, which are transcribed or encoded in an NGS data set. All contigs output by assembly tools were compared (BLAST) against reference genes for identifying gene segments.

To quantify the completeness, correctness, and length of gene segments, we propose four metrics: gene coverage, chimera rate, contig length, and contig coverage. For one reference gene, gene coverage is defined as the fraction of bases in the reference gene covered by at least one gene segment. The chimera rate is defined as the fraction of chimeric gene segments among all gene segments recovered by assembled contigs. As read mapping results can be used to determine the origin of each read, we can evaluate whether a gene segment is chimeric by checking the origin of each read in the gene segment. Contig coverage is the length of the gene segment normalized by the length of the target gene. This normalized contig length is used to prevent gene segments of long reference genes from dominating the average contig coverage of an assembly tool. Moreover, to evaluate the performance of assembly tools on recovering complete transcripts for RNA-Seq data using single contigs, we calculate the percentage of single contigs that can recover complete gene isoforms as well as the percentage of non-chimeric contigs among them. These experimental results can be found in the section of Performance of Recovering Gene Isoforms.

In Fig. 5, the length of gene 1 is 1000 bp and the combined length of the gene segments from contig 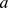 and contig 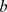 is 700 bp. Therefore, the gene coverage of gene 1 by contigs 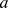 and 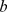 is 70%. The contig coverages of the two gene segments are 40% and 30% respectively. Suppose a bulk assembly tool outputs contig 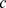, which covers both gene 1 and gene 2. Contig 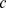 thus contains two gene segments. If 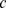 is a correctly assembled contig, it will generate 100% gene coverage for both gene 1 and gene 2. And, the chimera rate is zero for contig 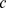 as the two gene segments are not chimeric. To compare the performance of assembly tools on all input families, we first calculate a metric for each family and then report the average of the values of the metric over all families.

### Gene assembly in an *Arabidopsis* RNA-seq data set

In this experiment, we applied SAT-Assembler to an RNA-Seq data set sequenced from a normalized cDNA library of *Arabidopsis* generated using paired-end Illumina sequencing [52]. There were a total of 9,559,784 paired-end reads of 76 bp. Pfam was used as our database of input families. Some Pfam families use sequences from *Arabidopsis* to train their profile HMMs. Therefore, we eliminated *Arabidopsis* sequences from these families and recomputed the profile HMMs for them. We compared the performance of SAT-Assembler with Velvet, Oases, Trinity, IDBA-Tran, and Trans-ABySS. Velvet is a widely used short read *de novo* assembly tool. Oases, Trinity, IDBA-Tran, and Trans-ABySS are assembly tools specially designed for transcriptomic data.

To determine which genes are transcribed in this data set, we conducted read mapping (using Bowtie [37]) on all the coding sequences (CDS) of *Arabidopsis thaliana* of version TAIR10 [53]. 59.62% of the input reads were mapped to the CDS with at most 2 mismaches allowed. There is no commonly accepted criterion to define transcribed genes. In this work, we defined CDS with at least 10 mapped reads as transcribed CDS. Assembly results of different tools were compared on these transcribed CDS. There are 29,030 different transcribed gene isoforms corresponding to 21,452 genes. A total of 3,163 protein or domain families from Pfam that can be aligned to these CDS using HMMER with gathering thresholds (GAs) were used as input to SAT-Assembler. Among the mapped reads, 65.39% generated HMMER hits against these protein or domain families using HMMER's default E-value threshold 10. The rest of the mapped reads failed to be aligned by HMMER due to the following main reasons: i) some *Arabidopsis* genes are not covered by Pfam families; ii) the average sequence identities of some Pfam families that Arabidopisis genes belong to are low, rendering marginal alignment scores, especially for short reads [47]; iii) some *Arabidopsis* genes are too remotely related to the Pfam families.

#### Edge creation performance

Before we evaluate the assembly performance of SAT-Assembler, we first evaluate the performance of the proposed edge creation strategy. We name the overlap threshold of SAT-Assembler *20+consistency* (or *consistency* for simplicity), where 20 is the default threshold for alignment overlap and consistency stands for the consistency between alignment overlap and sequence overlap. We compared edge creation performance of three strategies that used different overlap thresholds: i) 20; ii) 38; iii) consistency. Three metrics were used in the comparison: the number of true positive edges (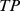), the number of false positive edges (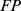), and positive predictive value (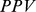), which is 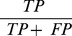. True positive edges connect nodes that are from the same genes. As the number of correct connections is the same for these strategies, 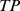 is proportional to the sensitivity. Higher 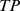 indicates higher sensitivity. False positive edges connect nodes that are from different genes. The performance comparison of three strategies is shown in Table 1.

**Table 1:** Edge creation performance of three strategies on the *Arabidopsis* RNA-Seq data set.

Strategy	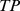	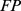	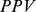
20	1,891,448	184,792	91.10%
38	1,658,268	13,469	99.19%
consistency	1,823,196	69,004	96.35%

The metrics are evaluated on all edges in overlap graphs of 3,163 families.

The consistency strategy provided a better trade-off for edge creation than the stringent overlap threshold of 38 and the very loose overlap threshold of 20. Compared with the 20 threshold strategy, the consistency strategy avoided 115,788 false positive connections at the expense of missing 68,252 positive connections, leading to 5.25% increase in 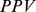. Compared with the 38 threshold strategy, it successfully captured 164,928 more positive connections, leading to 9.94% increase in 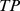 at the expense of 2.84% decrease in 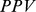. This showed that the consistency constraint successfully eliminated a large number of random overlaps while preserving overlaps between many reads from the same genes.

The conservation between transcripts and the input gene family will affect the performance of edge creation. When the conservation is good, the alignment overlap and sequence overlap are generally consistent. Therefore, the consistency strategy is able to capture the true overlaps between reads that are sequenced from the same transcripts. When the conservation between transcripts and the input family is poor, the alignment positions become less accurate. Thus, the two types of overlaps may not be consistent, making the reads from these transcripts fail to be connected by our consistency strategy. This is one main reason that the consistency strategy generated fewer 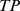 cases than the overlap threshold of 20 in Table 1. In our future work, we will incorporate the conservation of genes in our edge creation strategy to increase the 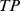 edges while maintaining the 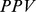 at a high level.

#### Performance comparison with other assembly tools

**Figure 6:**
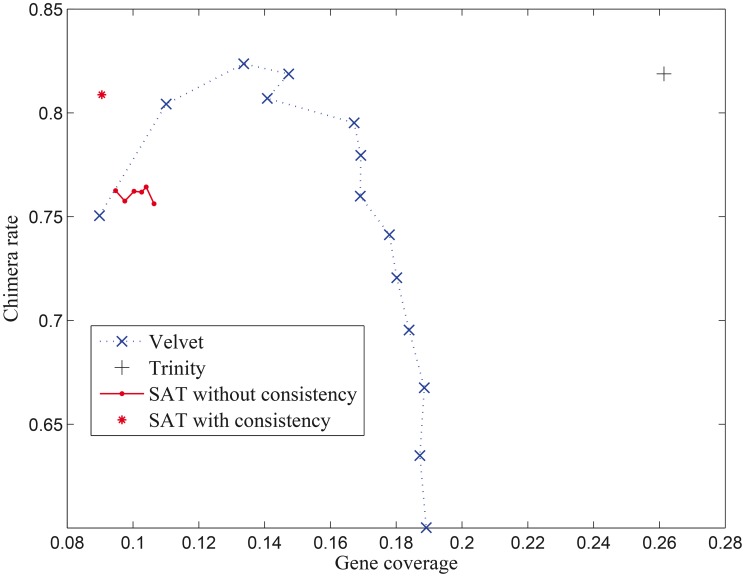
Chimera rate versus gene coverage. Chimera rate versus gene coverage when *k-mer* size or overlap threshold changes for different assembly tools. These values are average values of the assemblers' performance on 3,163 input families.

Velvet takes a single *k-mer* value for *de novo* assembly. Therefore, we tried *k-mer* sizes from 35 to 61 with a step size of 2. Oases, IDBA-Tran, and Trans-ABySS accept a range of *k-mer* sizes. All of them first generate assemblies on different *k-mer* sizes and then merge them. We ran them with a range of *k-mer* sizes from 35 to 61 with a step size of 2. Trinity uses a fixed *k-mer* size of 25 in its current implementation. We ran it with its default parameters. For SAT-Assembler, we used "consistency" as its edge creation strategy and default values for the other parameters. To compare the consistency strategy with the simple overlap threshold strategy, we also ran SAT-Assembler with an overlap threshold from 35 to 45 with a step size of 2 without the consistency constraint. Fig. 6 shows chimera rate versus gene coverage when we changed *k-mer* sizes or overlap thresholds for Velvet, Trinity, and SAT-Assembler. Oases, IDBA-Tran, and Trans-ABySS use a range of *k-mer* sizes and their performance is compared in Table 2.

**Table 2:** Performance comparison between different assembly tools on the *Arabidopsis* RNA-seq data set.

Tool	Velvet	Oases	Trinity	IDBA-Tran	Trans-ABySS	SAT
Gene coverage	82.36%	81.12%	81.88%	81.81%	78.31%	80.87%
Chimera rate	13.36%	29.24%	26.14%	21.74%	16.41%	9.05%
Contig length (bp)	346.89	455.75	418.62	450.64	355.08	434.24
Contig coverage	63.25%	84.61%	71.64%	78.19%	62.59%	76.65%
Memory (MB)	8034	22475	34691	3770	4427	2431
Time (m)	140.85	5128.74	1145.05	2774.73	3593.06	215.39

The memory usage for all tools is based on a single overlap threshold or *k-mer* and is evaluated as the peak memory usage of the tools. The running time was the average running time on all input families. SAT represents SAT-Assembler here and in all tables hereafter.

Velvet was sensitive to the change of *k-mer* sizes. The largest and smallest gene coverage of Velvet were 82.36% and 60.01% respectively. Its best overall performance was achieved when the *k-mer* size was 39. We also ran VelvetOptimiser [54] to search for the best assembly result by trying *k*-mer sizes from 35 to 61 bp with “
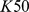
” as the optimization function. It reported an optimal *k-mer* size of 57, which generated a gene coverage of 66.39% and a chimera rate of 19.74%. The performance of SAT-Assembler was stable when the simple overlap threshold was changed. Overall, using the consistency strategy showed better gene coverage while keeping similar chimera rate compared with the simple overlap threshold.

We further compared the metrics of gene coverage, chimera rate, contig length, contig coverage, memory usage, and running time of different assembly tools when their best performance of gene coverage and chimera rate were achieved in Table 2. Contigs generated by all assembly tools covered about 80% of genes on average. SAT-Assembler had the lowest chimera rate due to our consistency-based edge creation strategy and graph traversal algorithm. Oases generated the longest contigs (indicated by contig length and contig coverage) on average. But the price paid was the highest chimera rate, longest running time, and second highest memory usage, even with a single *k-mer* size. As users will likely apply a range of *k-mer* sizes, its memory usage will be further increased. SAT-Assembler had the lowest memory usage and second shortest running time because of the effective classification in the homology search stage. The memory usage and running time of SAT-Assembler include both stages of homology search and *de novo* assembly. Our current implementation of SAT-Assembler uses the Python library of Networkx [55], which contributes to a large portion of both memory usage and running time. We plan to implement SAT-Assembler using C++ in the future.

#### Performance of recovering gene isoforms

The metric of gene coverage in the section of Performance Comparison with Other Assembly Tools shows the overall completeness of assembled contigs in recovering gene isoforms. Some gene isoforms with high gene coverage may be recovered by multiple contigs, which is not desirable for targeted gene assembly. In this section, we focus on the performance of assembly tools on producing contigs that recover complete gene isoforms of *Arabidopsis*. If a single contig recovers more than 90% of a gene isoform, it is defined as a *complete contig*. To compare the ability of different assembly tools in producing complete contigs, we evaluated the percentage of complete contigs generated by different assembly tools. Moreover, to evaluate how accurately these complete contigs recovered gene isoforms, we calculated the percentage of non-chimeric contigs among complete contigs. Table 3 shows the values of both metrics for different assembly tools.

**Table 3:** Performance comparison of different assembly tools on recovering complete gene isoforms of *Arabidopsis*.

Metric	Velvet	Oases	Trinity	IDBA-Tran	Trans-ABySS	SAT
Percentage of complete contigs	54.76%	80.17%	70.40%	74.73%	65.22%	73.93%
Percentage of non-chimeric contigs among complete contigs	79.12%	70.37%	75.65%	79.03%	77.97%	85.78%

Oases generated the highest percentage of complete contigs, which was consistent with its high gene coverage and contig length. Velvet had the lowest percentage of complete contigs, showing that it generated a lot of short gene segments. All the other tools had a percentage of complete contigs around 70%. IDBA-Tran had a good overall performance for recovering complete gene isoforms with 74.73% as the percentage of complete contigs and 79.03% as the percentage of non-chimeric contigs. SAT-Assembler had the highest percentage of non-chimeric contigs with 73.93% of its contigs as complete contigs. Its percentage of complete contigs is 0.8% lower than that of IDBA-Tran and its percentage of non-chimeric rate is 6.75% higher than that of IDBA-Tran. Because some true gene reads did not pass the homology search stage, some contigs generated by SAT-Assembler were segmented and not able to recover complete gene isoforms. However, it had the best performance on distinguising gene isoforms due to AS events. We plan to increase the sensitivity of the homology search stage using strategies such as PSST [47], [56] to achieve better overall performance of recovering gene isoforms for RNA-Seq data.

### Targeted gene assembly in a metagenomic data set from synthetic communities

In this experiment, we conducted targeted gene assembly using a metagenomic data set sequenced from highly diverse *bacterial* and *archaeal* synthetic communities with 16 *archaea* members and 48 *bacteria* members [57]. We downloaded all reference genomes from NCBI ftp site (ftp.ncbi.nih.gov/genomes/). The metagenomic data set was downloaded from NCBI Sequence Read Archive (SRA) (http://www.ncbi.nlm.nih.gov/sra) using Accession No. SRA059004. After we trimmed low-quality reads, there were 51,933,622 paired-end reads with an average read length of 100 bp. We were interested in assembling the family of butyrate kinase pathway genes, which play important roles in butyrate synthesis. We downloaded the profile HMM of the family from RDP's functional gene repository [58]. It was built from 77 seed butyrate kinase pathway genes. The seed genes are not in the genomes. We annotated the butyrate kinase gene regions in the genomes by aligning reference genomes against the gene family using HMMER with gathering thresholds (GAs). We compared the performance of Velvet, IDBA-UD, MetaVelvet, and SAT-Assembler on assembling contigs from these regions. IDBA-UD and MetaVelvet are both de Bruijn graph based and specially designed for *de novo* assembly of metagenomic data.

We used VelvetOptimiser to search for the best assembly result by trying *k-mer* sizes from 53 to 83 bp with “
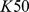
” as the optimization function. VelvetOptimiser reported 55 as the optimal *k-mer* size. For IDBA-UD, which accepts multiple *k-mer* values, we used the same range of *k-mer* sizes as Velvet in a single run. Meta-Velvet used the hash table generated by Velvet and its *k-mer* size was thus 55 as well. For SAT-Assembler, we used its default parameters. A total of 15,254 reads were classified into the butyrate kinase family by the homology search stage, which accounted for 0.15% of the query reads. Table 4 shows a performance comparison between these assembly tools.

**Table 4:** Performance comparison between different assembly tools in assembling genes from butyrate kinase family on the synthetic metagenomic data set.

Assembly tool	Velvet	IDBA-UD	MetaVelvet	SAT-Assembler
Gene coverage	68.17%	75.08%	79.68%	88.37%
Chimera rate	26.31%	12.54%	16.12%	8.43%
Contig length (bp)	367.89	592.29	440.94	487.68
Contig coverage	35.81%	56.62%	42.35%	46.69%
Memory usage (MB)	41404	15162	18340	307
Time (m)	1262.87	956.38	817.11	1093.86

The memory usage for all tools is based on a single overlap threshold or *k-mer* and is evaluated as the peak memory usage of the tools. The running time was the average running time on all input families.

SAT-Assembler had the best gene coverage, chimera rate, and memory usage. Its contigs were usually shorter than IDBA-UD. A closer examination reveals the reason: a number of reads did not pass the profile HMM homology search and thus were not used as input to assembly. Gene coverage of assembly tools in this experiment was much lower than in the first experiment because of lower sequencing depth and higher data complexity. MetaVelvet had the best running time performance because it directly used the optimal *k-mer* size and hash table from VelvetOptimiser while Velvet and IDBA-UD both ran a range of *k-mer* sizes. The low memory usage of SAT-Assembler further showed the advantage of using homology search in targeted gene assembly for large-scale NGS data. Due to the high complexity of the metagenomic data set, SAT-Assembler constructed a much more complex overlap graph compared with the overlap graphs in the first experiment, leading to higher runtime overhead. Table 4 shows that none of the tested assembly tools is the best in all metrics. If users prefer high gene coverage and high accuracy, especially on hardware with limited resources, we recommend SAT-Assembler. If long contigs and high contig coverage are more important, IDBA-UD is the best choice.

### Targeted gene assembly in a human gut metagenomic data set

In this experiment, we compared the performance of SAT-Assembler with Velvet, IDBA-UD, and MetaVelvet on a human gut metagenomic data set. There were 47,117,906 paired-end and 5,528,102 unpaired reads of various lengths. The average length of the query reads was 95.72 bp and 75% of them were 100 bp. We were interested in assembling butyrate kinase pathway genes as in the second experiment. The profile HMM of the gene family was built from 77 seed genes from RDP's functional gene repository [58]. We also downloaded a set of 2,352 annotated genes of butyrate kinase family and eliminated the seed genes from them. By using read mapping, a total of 58 genes with at least 10 mapped reads were identified and were used to evaluate the performance of all assembly tools.

We used VelvetOptimiser to search for the best assembly result by trying *k-mer* sizes from 51 to 81 bp with “
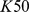
” as the optimization function. VelvetOptimiser reported 51 as the optimal *k-mer* size. For IDBA-UD, which accepts multiple *k-mer* values, we used the same range of *k-mer* sizes as Velvet in a single run. Meta-Velvet used the hash table generated by Velvet and its *k-mer* size was thus 51 as well. For SAT-Assembler, we used its default parameters. A total of 16,136 reads were classified into the butyrate kinase family by the homology search stage, which accounted for 0.31% of the query reads. Table 5 shows a performance comparison between these assembly tools.

**Table 5:** Performance comparison between different assembly tools in assembling genes from butyrate kinase family on the human gut metagenomic data set.

Assembly tool	Velvet	IDBA-UD	MetaVelvet	SAT-Assembler
Gene coverage	51.97%	60.55%	43.52%	68.89%
Chimera rate	59.26%	36.67%	36.73%	16.87%
Contig length (bp)	505.70	738.64	672.66	661.35
Contig coverage	38.99%	62.06%	49.30%	48.56%
Memory usage (MB)	41248	16648	20330	283
Time (m)	1736.08	1065.77	834.10	1530.63

The memory usage for all tools is based on a single overlap threshold or *k-mer* and is evaluated as the peak memory usage of the tools. The running time was the average running time on all input families.

In this experiment, the result of performance comparison was similar to the second experiment. SAT-Assembler still had the best gene coverage, chimera rate, and memory usage. IDBA-UD had the best contig length and contig coverage. Compared with the second experiment, the chimera rates of all assembly tools increased. Without knowing all reference genomes, the computed chimera rates might be an over-estimation for all tools because the assembled contigs may contain novel members of the family.

## Discussion

The experiments on RNA-Seq and metagenomic data sets show that our novel consistency-based edge creation strategy and guided graph traversal can effectively avoid chimeric contigs. Moreover, by reducing the original search space into a much smaller subset of reads from targeted genes, the memory usage was significantly decreased, making it a more economical tool for the assembly of targeted genes from a single or multiple pathways. We have also tried to use Velvet and Trinity on the reads that passed the homology search stage on the *Arabidopsis* RNA-Seq data set. The gene coverage and chimera rate of Velvet were 64.38% and 17.25% respectively. The gene coverage and chimera rate of Trinity were 78.72% and 22.90% respectively. Compared with the performance when using Velvet and Trinity directly on the input data set, their gene coverages were decreased. One reason is that the homology search stage does not have 100% sensitivity. The missed reads may lead to poorer performance of Velvet and Trinity.

SAT-Assembler also provides an easier way for users to set the parameters. Our edge creation strategy is based on both the overlap threshold (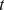) and the consistency between alignment overlap and sequence overlap (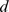). The consistency strategy poses a strong constraint on the overlap between two reads. The alignment overlap threshold 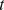 is mainly used to avoid random overlaps, which are generally very small. The default overlap threshold 20 is chosen based on the length of the reads in our experimental data sets. This value is smaller than the *k-mer* value chosen by VelvetOptimiser for other assembly tools. This helps us generate better connectivity between reads from the same genes. At the same time, the consistency constraint guarantees the accuracy of the edge creation (Table 1). We have also tried different 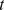 values from 15 to 30 and found that the edge creation performance is not sensitive to the choice of 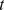 unless a very large value of 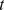 is used. The values of 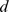 and 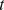 control the trade-off between sensitivity and 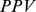 of edge creation. Users can adjust them based on their specific need. Based on our observation, an overlap threshold that is 20% of the read length is recommended. The value of 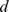 is independent of the read length and we suggest that users use the default value.

There are still some challenges to address to further improve SAT-Assembler's performance. First, gene segments from some poorly conserved gene regions are fragmented because some reads from these regions fail to pass the homology search. We have aligned all the reads in the human gut metagenomic data set against protein/domain families in Pfam using HMMER and 38.65% of them have HMMER hits. There are three main reasons for the low coverage of Pfam domains in the metagenomic data set: i) Pfam is a collection of protein/domain families. Therefore, reads sequenced from intergenic regions will not have hits. In addition, even reads sequenced from protein-coding regions may not be part of any domain. They will not have hits either. ii) Some genes of the microbial species are very remotely homologous to the families in Pfam. iii) Some reads in the metagenomic data set are very short, resulting in low sensitivity of HMMER [47]. This problem can be alleviated by increasing the sensitivity of the homology search. In the future, we will incorporate our proposed position-specific score threshold (PSST) [47], [56] into SAT-Assembler to classify more reads into their native families.

**Figure 7:**
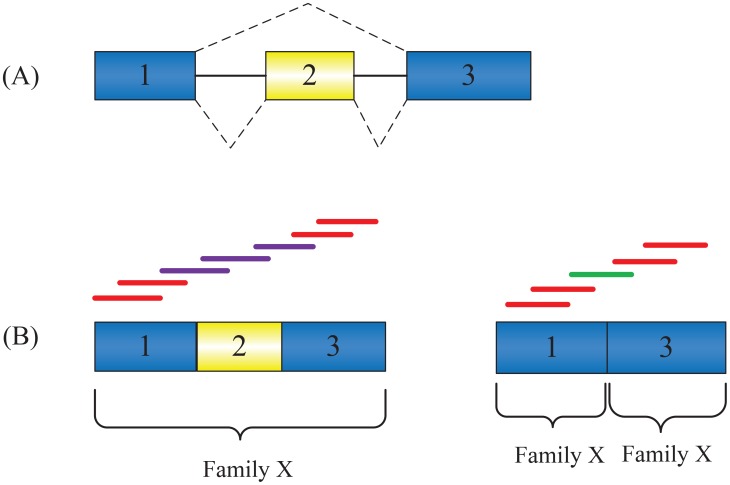
Poor conservation can lead to loss of edge connection. (A) Gene isoforms due to AS events. Constitutive exons are shown in blue and alternatively spliced regions in yellow. Introns are represented by solid lines, and dashed lines indicate splicing options. (B) Two gene isoforms and their sequenced reads. The red lines represent reads sequenced from exons shared by both isoforms. The purple and green lines represent reads that are exclusively sequenced from each isoform. Both isoforms can be aligned with Family X. The isoform containing exons 1, 2, and 3 has a global alignment while the isoform containing exons 1 and 3 produces two local alignments.

Second, although the edge creation strategy of SAT-Assembler captured more overlaps between reads from the same genes, some positive overlaps still failed to be captured. When the conservation between the input family and target genes is not good, the alignment overlap and sequence overlap may not always be consistent. Therefore, reads from poorly conserved regions of the genes may lose consistency between their alignment overlaps and sequence overlaps. In this case, connectivity between these reads will not be captured by SAT-Assembler's edge creation strategy, leading to segmented contigs in the final assemblies. Fig. 7 shows an example of missing edge connection due to poor conservation between gene isoforms and the input family. Both gene isoforms are from the same family X. The first gene isoform has a global alignment against Family X while the skipped exon 2 in the second gene isoform leads to two local alignments. According to our edge creation strategy, all the reads in the first gene isoform can be correctly connected. However, because the green read shares sequence overlap but no alignment overlap with neighboring reads, there will be no edge between the green read and its neighboring reads, leading to two disconnected contigs. In this case, the two contigs can usually be connected in our scaffolding stage using paired-end reads. As part of our future work, we will take into consideration the conservation between target genes and the family to improve our edge creation strategy. Moreover, insertion/deletion (indel) or substitution errors in the overlapping regions may also lead to false negative connections. Masella et al. [59] proposed a sophisticated method that can probabilistically correct these errors based on the overlap data from the paired-end reads. HMM-FRAME [60] can be used to accurately detect and correct indel errors using profile HMM-based homology search. We plan to incorporate these methods in our edge creation strategy to generate more positive connections.

Third, the running time of the graph traversal stage is the bottleneck of SAT-Assembler, especially for complex metagenomic data. Therefore, we plan to add more bounding strategies into the graph traversal, such as a more stringent threshold for critical supports. Moreover, we will implement SAT-Assembler using C++ to reduce its running time.

## Supporting Information

Text S1 **Supplementary material.** Pseudocodes and experimental settings.
(PDF)
