# Supplementary material for: A Scalable and Accurate Targeted Gene Assembly Tool (SAT-Assembler) for Next-Generation Sequencing Data
Source: PLoS Comput Biol. 2014 Aug 14;10(8):e1003737. doi: 10.1371/journal.pcbi.1003737 (PMC4133164; doi:10.1371/journal.pcbi.1003737)
Supplement: Text S1 — Supplementary material. Pseudocodes and experimental settings. (PDF) [file pcbi.1003737.s001.pdf]

Supplementary Material:
A Scalable and Accurate Targeted gene Assembly tool (SAT-Assembler) for next-generation sequencing data

Yuan Zhang[1], Yanni Sun[1,*], James R. Cole[2]
1 Department of Computer Science and Engineering, Michigan State University, East Lansing, MI, 48824
2 Center for Microbial Ecology, Michigan State University, East Lansing, MI, 48824
∗ **E-mail: yannisun@msu.edu**

1 Pipeline of SAT-Assembler

In this document, we present the pseudo-codes of five stages of SAT-Assembler.

1. Profile HMM-based homology search
2. Alignment-informed graph construction
3. Pruning and optimization of overlap graphs
4. Guided traversal using multiple types of information
5. Contig scaffolding

2 Pseudocodes of SAT-Assembler

Procedure 1 Profile HMM-based homology search

Input: S: input sequences; M: target gene families; p: the E-value threshold for HMMER.
Output: H: sequences that pass the homology search.

for each target gene family M_i **do**
  align S against M_i
  add sequences that generate E-value $\leq p$ into H_i
end for
for each sequence s in H **do**
  assign s to up to three families that generate the best E-values and update H
end for
return H

Procedure 2 Alignment-informed graph construction

Input: H: a list of N reads sorted by their alignment beginning positions; t^*: the alignment overlap threshold; d^*: relative difference threshold.
Output: G: the overlap graph.

```
//add vertices to G
for i = 1 → N do
    create a vertex V_i
    add V_i to G
end for
//add edges to G
for i = 1 → N − 1 do
    for j = i + 1 → N do
        t =the alignment overlap between V_i and V_j
        k =the sequence overlap between V_i and V_j
        if t ≥ t* and |t − k|/t ≤ d* then
            create an edge E_{i,j}
        else if t ≤ t* then
            break
        end if
    end for
end for
return G
```

Procedure 3 Pruning and optimization of overlap graphs

Input: G: the overlap graph.
Output: G: the overlap graph after pruning and optimization.

```
//remove transitive edges
for each edge in G ⟨V_i, V_j⟩ do
  if there is a transitive edge between V_i and V_j then
    remove ⟨V_i, V_j⟩
  end if
end for
//simplify the overlap graph
while there exists a node that has a single outgoing edge and its successor has a single incoming edge do
  merge these two nodes. Update their corresponding in-edges and out-edges appropriately.
end while
//remove tips
for each node v in G do
  if the in-degree or out-degree of v is zero and its coverage is less than 2 then
    remove v
  end if
end for
//remove redundant edges
find all rectangles R in G
for path p in R do
  if there is another path that has the same starting and ending nodes and the coverage is higher than p then
    remove p
  end if
end for
```

Procedure 4 Guided graph traversal using multiple types of information

Input: G: the overlap graph; t: threshold for critical support.
Output: C: contigs.

```
for node v in nodes that have zero in-degree do
  p = an empty path
  DFS(G, v, t, p, C)
end for
```

Procedure 5 DFS(G, v, t, p, C)

Input: G: the overlap graph; v: the current node; p: the current path; t: the threshold for critical support.
Output: C: the contigs.

```
add v into p
//evaluate critical support when a non-chimeric node is added.
if v has zero out-degree then
  generate the contig from p and add it into C.
else if v is a non-chimeric node and the critical support of p is below t then
  generate the contig from p and add it into C.
  for v∗: each successor of v do
    p* = an empty path.
    DFS(G, v*, p*, t, C)
  end for
else
  DFS(G, v*, p, t, C)
end if
```

Procedure 6 Contig scaffolding

Input: C: N contigs.
Output: S: scaffolds.

```
//use an indirect graph to keep paired-end reads between contigs.
for i = 1 → N do
  create a node for each contig
end for
create a undirected graph H.
for i = 1 → N − 1 do
  for j = 2 → N do
    if there are paired-end reads between C_i and C_j then
      create an edge ⟨v_i, v_j⟩ in H for C_i and C_j
    end if
  end for
end for
return all connected components in H
```

3 Experimental data sets and settings

3.1 Experiment on the *Arabidopsis* RNA-Seq data set

- The data set is archived in Short Read Archive (SRA) (`http://www.ncbi.nlm.nih.gov/sra`) under the accession number SRA047499.
- The HMM files can be downloaded from Pfam website: `ftp://ftp.sanger.ac.uk/pub/databases/Pfam/releases/Pfam27.0/Pfam-A.hmm.gz`.
- The command used to run Velvet is: VelvetOptimiser-2.2.5/VelvetOptimiser.pl -s 51 -e 51 -f '-fasta -shortPaired -separate SRR360147.1.fasta SRR360147.2.fasta' -t 4 –optFuncKmer 'n50'

- The command used to run Oases is: ./oases_pipeline.py -m 49 -M 61 -o Result -d "-fasta -shortPaired -separate SRR360147.1.fasta SRR360147.2.fasta" -p "-ins_length 350"
- The command used to run Trinity is: ./Trinity.pl –seqType fa –left SRR360147.1.fasta –right SRR360147.2.fasta –JM 50G –output Trinity
- The command used to run IDBA-Tran is: idba_tran -r SRR360147.both.fasta –mink 35 –maxk 61 –step 2 -o out_dir
- The command used to run Trans-ABySS is to first run ABySS on a range of *k-mers*: abyss-pe name=arabidopsis n=10 k=$kmer in='SRR360147_1.fastq SRR360147_2.fastq', where *kmer* is a variable from 35 to 61. We then ran trans-abyss pipeline on the assembled contigs.
- The command to run SAT-Assembler is: ./SAT-Assembler.sh -m all_families.hmm -f SRR360147.both.fasta -o out_dir.

3.2 Experiment on the metagenomic data set of synthetic communities

- The data set is archived in SRA under the accession number SRA059004.
- The HMM model of family of butyrate kinase pathway genes can be downloaded from RDP's functional gene repository: `http://fungene.cme.msu.edu/hmm_download.spr?hmm_id=310`.
- The command used to run Velvet is: VelvetOptimiser-2.2.5/VelvetOptimiser.pl -s 53 -e 83 -x 2 -f "-fasta -shortPaired -separate SRR606249_end1.fasta SRR606249_end2.fasta" -t 4 –optFuncKmer 'n50'
- The command used to run IDBA-UD is: idba_ud -r SRR606249_both.fasta –mink 53 –maxk 83 –step 2 -o output_dir
- To run Meta-Velvet, we used the following steps: 1) ran velveth: velveth Result 55 -fasta -shortPaired -separate SRR606249_end1.fasta SRR606249_end2.fasta; 2) ran velvetg: velvetg Result -exp_cov auto -ins_length 260; 3) ran MetaVelvet: meta-velvetg Result -ins_length 260
- The command used to run SAT-Assembler is: ./SAT-Assembler.sh -m buk_rdp.hmm -f SRR606249_both.fasta -o Result

3.3 Experiment on the human gut metagenomic data set

- The data set can be downloaded at: `ftp://public-ftp.hmpdacc.org/Illumina/stool/SRS015217.tar.bz2`.
- The HMM file is the same as in the second experiment.
- The command used to run Velvet is: VelvetOptimiser-2.2.5/VelvetOptimiser.pl -s 51 -e 51 -f '-fasta -shortPaired -separate SRS015217.1.changed.fa SRS015217.2.changed.fa -fasta -short2 SRS015217.single.changed.fa' -t 4 –optFuncKmer 'n50'
- The command used to run IDBA-UD is: idba_ud -r SRS015217.merged.fa -o output –mink 51 –maxk 81 –step 2
- To run Meta-Velvet, we used the following steps: 1) ran velveth: velveth Result 51 -fasta -shortPaired -separate SRS015217.1.fa SRS015217.2.fa -fasta -short2 SRS015217.single.fa; 2) ran velvetg: velvetg Result -exp_cov auto -ins_length 260; 3) ran MetaVelvet: meta-velvetg Result -ins_length 260

- The command used to run SAT-Assembler is: ./SAT-Assembler -m buk_rdp.hmm -f SRS015217.all.fa -o Result
